# Some Dangers of Spoon-Feeding

**Published:** 1913-03-08

**Authors:** Binnie Dunlop

**Affiliations:** Alexandra Court, S.W.


### Some Dangers of Spoon-feeding.

*To the Editor of* The Hospital.

Sir,—I am glad to see The Hospital supporting Mr. Geoffrey Drage's most timely demand that the Government should order a return to be prepared of the total expenditure out of national and local funds on State charity. Yet it ought not to need such a return to convince us that a very considerable section of the community is living and, what is much more serious, propagating, at the expense largely of the rest of the community; that people are being allowed—nay, encouraged—to bring more children into the world than they can even feed sufficiently. How is this grave parental irresponsibility to be checked? We cannot adopt a *laissez-faire* attitude and leave these necessitous children to starve and die. We cannot maintain them and later (even if we trained them to skilled work) find full-wage jobs for them. After much study of this problem I have come to the conclusion that there is only one way of solving it. We must *encourage* the poor not to beget children they cannot provide for. There are most serious troubles in store for all classes if hospitals, doctors, midwives, and nurses do not immediately begin to teach the poor the hygienic means of family limitation. It is both cruel and dangerous to go on ignoring that comparatively few wage-earners can do justice to more than two children, that many feeble-minded children and "Weary Willies" would never have been born if their parents had known better, and that the growing burden of State charity is forcing the thrifty and industrious more and more to restrict their own numbers.—Yours faithfully,

Binnie Dunlop, M.B., Ch.B.

Alexandra Court, S.W.
March 1, 1913.

### The Prolongation of Useless Lives.

*To the Editor of* The Hospital.

